# The *Mycobacterium tuberculosis* genome at 25 years: lessons and lingering questions

**DOI:** 10.1172/JCI173156

**Published:** 2023-10-02

**Authors:** Benjamin N. Koleske, William R. Jacobs, William R. Bishai

**Affiliations:** 1 Center for Tuberculosis Research, Department of Medicine, Johns Hopkins School of Medicine, Baltimore, Maryland, USA.; 2 Department of Microbiology and Immunology, Albert Einstein College of Medicine, Bronx, New York, USA.

## Abstract

First achieved in 1998 by Cole et al., the complete genome sequence of *Mycobacterium tuberculosis* continues to provide an invaluable resource to understand tuberculosis (TB), the leading cause of global infectious disease mortality. At the 25-year anniversary of this accomplishment, we describe how insights gleaned from the *M. tuberculosis* genome have led to vital tools for TB research, epidemiology, and clinical practice. The increasing accessibility of whole-genome sequencing across research and clinical settings has improved our ability to predict antibacterial susceptibility, to track epidemics at the level of individual outbreaks and wider historical trends, to query the efficacy of the bacille Calmette-Guérin (BCG) vaccine, and to uncover targets for novel antitubercular therapeutics. Likewise, we discuss several recent efforts to extract further discoveries from this powerful resource.

## Introduction

With 10.6 million active cases and 1.6 million deaths annually, tuberculosis (TB) causes greater mortality than any other single pathogen (1). Treatment regimens for TB require months of therapy with high rates of associated toxicities and rising drug resistance (1, 2). From 1985 to 1992, the TB incidence rate in the United States increased for the first time after decades of decline, an event that stimulated a new era of intensive research on TB (3). In 1998, Stewart Cole at the Institut Pasteur and colleagues at the Sanger Centre in the United Kingdom published the first complete genome sequence of a *Mycobacterium tuberculosis* strain, the virulent laboratory reference strain H37Rv (4). While the genome sequence was already transformative at the time, the past 25 years of progress have substantially increased its impact on TB taxonomy, drug discovery, resistance mechanisms, epidemiology, vaccine development, and pathogenesis.

Whole-genome sequencing (WGS) of *M. tuberculosis* and related mycobacteria is now routine, allowing comparisons across time and space. This increased capability has enabled epidemiological studies at the local and global levels to track TB outbreaks and discover features suggestive of increased transmissibility (5–7). Likewise, diagnostics for multi-drug-resistant (MDR) and extensively drug-resistant (XDR) strains make heavy use of knowledge gleaned from *M. tuberculosis* genome sequencing (8, 9). The recent development of novel antitubercular drugs, including bedaquiline, pretomanid, and delamanid, shows how genomics can be used to understand bacterial susceptibilities to therapy (10, 11). Beyond pathogenic *M. tuberculosis* strains, sequencing of the live attenuated bacillus Calmette-Guérin (BCG) vaccine for TB has increased recognition of global vaccine heterogeneity, with potential implications for patient immunity and studies to improve vaccination (12–15).

The *M. tuberculosis* genome has ushered in a quarter century of substantial clinical and public health advancements. Alongside gene transfer, the vital technology enabling manipulation of mycobacterial DNA, these discoveries built the foundation for much modern *M. tuberculosis* work (16–27). For example, greater sequencing depth has uncovered bacterial polymorphisms whose roles in host adaptation and antimicrobial resistance remain under investigation (28–31). In addition, temporal studies have identified *M. tuberculosis* heterogeneity within individual patients and have demonstrated a progressive evolutionary process leading to drug resistance at the population level (32–36). Lastly, improvements in genome-wide screening studies have shown promise in identifying candidate pathways to target therapeutically (37–39). We comment here on what has already been achieved as a result of the *M. tuberculosis* genome sequence and also explore what more the *M. tuberculosis* genome may yield.

## *M. tuberculosis* lineages

**Figure 1:**
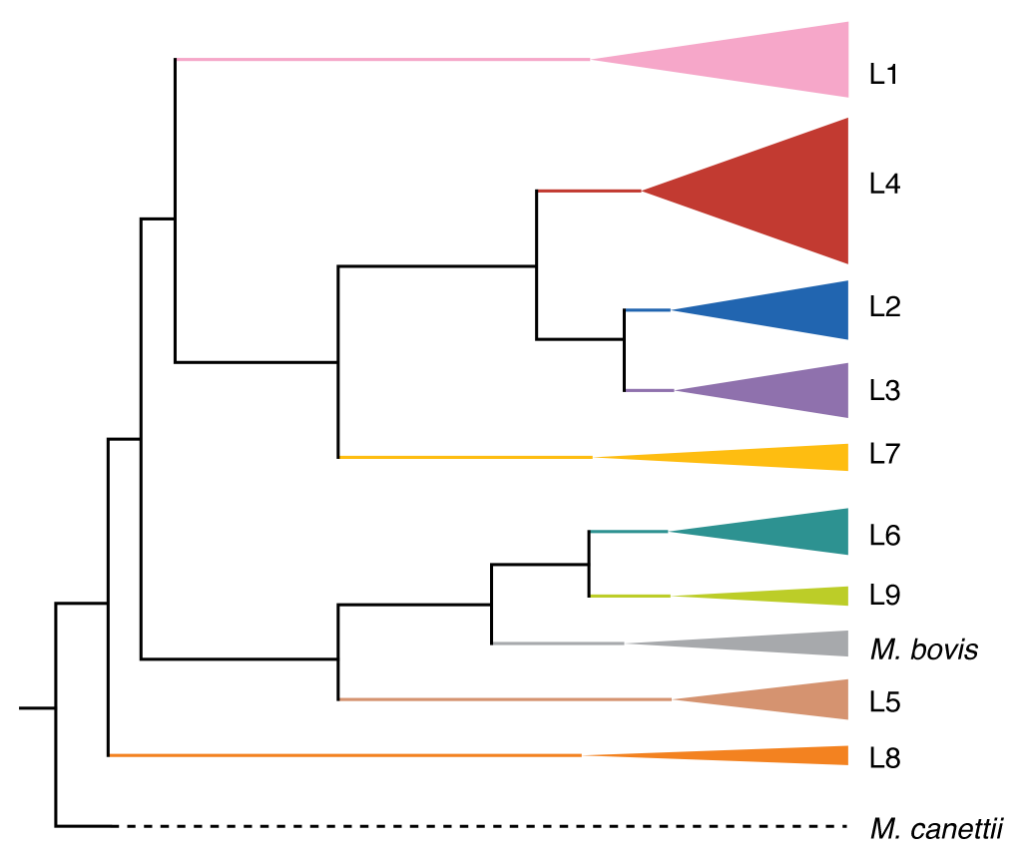
Phylogeny of *M. tuberculosis* lineage strains. Simplified maximum likelihood phylogeny of the 9 lineages of *M. tuberculosis*, as well as the related *M. bovis* strain and the *M. canettii* outgroup strain used as a root. Adapted with permission from *Microbial Genomics* (60).

Not long after the first *M. tuberculosis* genome sequence was assembled, additional studies revealed the global diversity of *M. tuberculosis* genotypes. Previous methods of typing *M. tuberculosis* strains, including IS6110-RFLP (restriction fragment length polymorphism), spoligotyping (spacer oligonucleotide typing, which is recognized as using CRISPR array spacers), and MIRU-VNTR (mycobacterial interspersed repetitive unit variable number tandem repeat) typing, had found some success using variable numbers or positions of repetitive genomic elements, although they were limited by the low abundance and occasional non-uniqueness of these markers (40, 41). Indeed, previous attempts to predict clinical strain parameters by IS6110 copy number were found to be suboptimal, as the so-called low-copy and high-copy groups were later shown to be polyphyletic (42, 43). In contrast, comparing single-nucleotide polymorphisms (SNPs) across the genome allowed for unambiguous, higher-resolution assignment of *M. tuberculosis* strains into lineage clusters (Figure 1) (41–43). Strikingly, the broad lineages of *M. tuberculosis* also clustered with the birthplaces of the patients they infected, suggesting that the *M. tuberculosis* phylogeny and indeed the *M. tuberculosis* genome had captured geographic information (43, 44). Comparing *M. tuberculosis* strains from diverse lineages showed a paucity of large genomic deletions and rearrangements, evidence of an exceptionally stable genome among bacteria that preserved historical information (44). By the turn of the century, the *M. tuberculosis* genome was recognized as a powerful spatiotemporal resource to understand the epidemiology of the disease.

### M.

**Figure 2:**
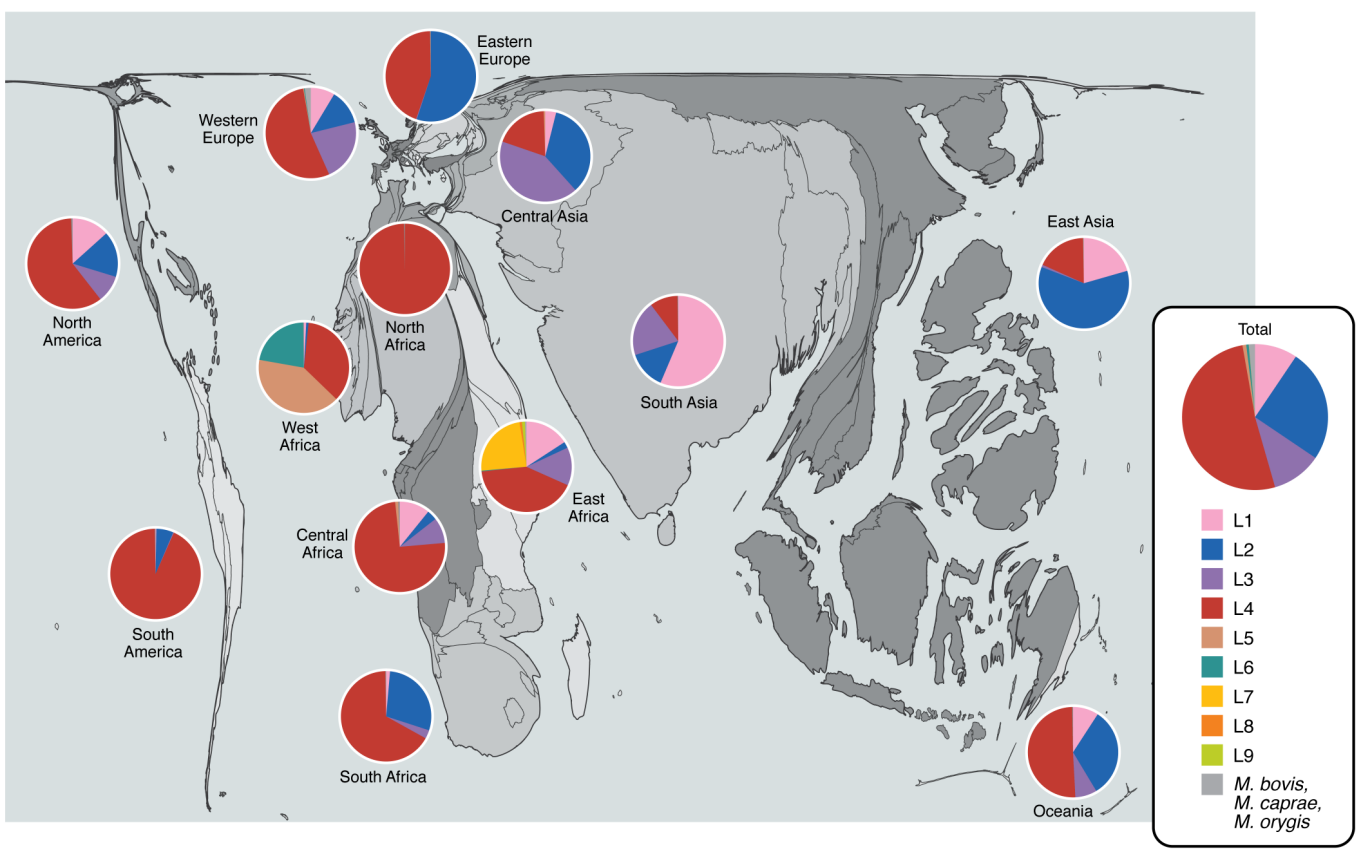
Cartogram of global TB burden by *M. tuberculosis* lineage. Country areas are scaled to reflect TB incidence in 2021 (1) using the go-cart.io algorithm (172). Pie charts reflect distributions of the *M. tuberculosis* lineages L1–L9, as well as the animal-adapted *M. bovis*, *M. caprae*, and *M. orygis*, from clinical isolates by geographic region as previously described by Napier et al. (56).

*tuberculosis* has been classified into nine lineages mainly on the basis of large-scale genomic variations, variably described as regions of difference (RDs) or large sequence polymorphisms (LSPs) (45, 46). Lineages 2–4 (L2–L4) constitute a monophyletic group defined by the TbD1 deletion, a loss of the membrane proteins MmpS6 and MmpL6 that appears to confer enhanced resistance to oxidative and hypoxic stressors (47, 48). Collectively termed the “modern” lineages, L2–L4 cause the majority of globally distributed TB epidemics and hence most of the TB disease burden (Figure 2) (49). Among these, L4 has the broadest range, spanning throughout Africa, Asia, Europe, and the Americas. L2 causes the largest proportion of TB cases in East Asia as well as some cases in Central Asia and notably includes the hypervirulent Beijing strains, while L3 exists mainly in India, with additional presence in East Africa. The remaining “ancestral” lineages include L1, which predominantly occurs in Southeast Asia and India and has the widest distribution of the ancestral lineages. L5–L9 seemingly arise only in Africa, with L5–L6 (classically dubbed *M. africanum*) causing up to 40%–60% of TB cases there (50). Notably, the animal-adapted lineages of the broader *M. tuberculosis* complex, including *M. bovis*, share ancestry with L6 (51). Beyond these overarching categories, the modern lineages can be divided into sublineages that also correlate with human population geography (52, 53). These sublineages can be further classified into “generalist” sublineages with broader global distribution and greater variability in T cell epitopes than the more geographically confined “specialist” sublineages (53). Consistent with these observations, recent evidence argues for sympatric spread among specialist sublineages, suggesting that specialist strains have adapted to the human host genetics in their endemic regions (54). Recent methods expand beyond the use of RDs and incorporate SNPs into a highly granular sublineage classification schematic (55, 56).

By integrating the diversity of modern *M. tuberculosis* genomes, attempts have been made to determine the origin of human-adapted TB disease and follow its evolution with changing human migration and behavior. The discovery of *Mycobacterium canettii* in the Republic of Djibouti and its subsequent genomic characterization as an *M. tuberculosis* ancestor localized the origin of ancient TB to an origin around the Horn of Africa (57–59). Clinically, *M. canettii* strains are of relatively low virulence, and their genomes are generally devoid of the RDs/LSPs that define lineages L1–L9 (46, 60). Solidifying an emergence in East Africa, a new ancestral lineage with very deep phylogenetic branching, L7, was found in Ethiopia in 2012 and appears limited to residents of and immigrants from the region (61). In the past few years, two more analogously restricted East African lineages (L8–L9) were characterized (60, 62). While horizontal gene transfer mechanisms are not believed to occur in modern *M. tuberculosis* genomes, the ancient genome contains a mosaic of genetic material, likely from nonpathogenic bacteria (59). Earlier efforts to sequence large *M. tuberculosis* genomic regions identified a comparatively low rate of silent nucleotide mutations in comparison with other human pathogens, suggesting a population bottleneck with *M. tuberculosis* adaptation to the parasitic lifestyle (63). These findings collectively suggest a model in which environmental bacteria supplied genomic material to what would become the obligate human pathogen *M. tuberculosis*, replacing historical arguments for a zoonotic origin (59, 64). From East Africa, *M. tuberculosis* would have spread globally alongside its human host populations, and indeed the phylogeny of human mitochondrial DNA shows similar topology to that of the *M. tuberculosis* lineages (64). Like its human hosts, *M. tuberculosis* underwent several population bottlenecks during geographic spread (65). These events were followed by periods of diversification featuring many non-synonymous SNPs, notably in cell envelope proteins that may have facilitated bacterial virulence by adapting to the host immune system (65, 66). Alterations in human population demographics correlate well with *M. tuberculosis* evolution on both the ancient time scale, such as the dissemination of the L2 Beijing sublineage as an agricultural lifestyle spread from China across East Asia 3,000–5,000 years ago, and the nearer time scale, such as the spread of this sublineage to Afghanistan during recent wars and the rise in TB drug resistance in former Soviet states with the collapse of the USSR (64, 67). These findings demonstrate the impressive power of the *M. tuberculosis* genome to record and adapt to host changes throughout human history. Simultaneously, the recent data in particular highlight the tendency of *M. tuberculosis* to exploit periods of social instability and forced migration to escalate into a greater public health threat.

The non-random expansion of particular *M. tuberculosis* lineages into global pandemics suggests that these strains may have altered properties relevant to disease outcomes, a hypothesis that echoes the validation of the TbD1 deletion as a gain-of-virulence event based on animal model studies (48). Relatedly, differing lineages have been found to elicit variable immune responses in cellular and animal model systems, and there is some evidence of differential immune modulation by the modern lineages related to these properties (68–73). L2, for example, has been found to induce lower levels of inflammatory cytokines than L4 in some but not all studies (68, 69, 74). However, care must be taken in synthesizing results between such studies, particularly in attempting to generalize results from individual strains across entire lineages. As an example, virulence factors may exist only among certain subgroups within a lineage, as is the case with the cell wall phenolic glycolipid (PGL) that facilitates immune evasion by the subsets of the Beijing sublineage that express it (75, 76). Consistent with this explanation, a comparatively modern Beijing strain was found to cause reduced cytokine secretion in a macrophage model when compared with a more ancestral Beijing strain, despite similarities in bacterial burden and growth rate (71). While all *M. tuberculosis* strains isolated from patients with active TB are virulent by definition, the observed differences in global transmissibility suggest subtler differences in virulence by lineage due to genome variations, as has been suggested by high-throughput studies (73).

## Antimicrobial resistance

The growing antimicrobial resistance of *M. tuberculosis* presents an enormous clinical, financial, and public health challenge (1). Genomic sequencing has shown considerable promise in predicting *M. tuberculosis* susceptibility to TB drugs, and gene transfer had enabled the discovery of resistance genotypes, thereby elucidating previously unknown mechanisms of action (27, 77). In a study of over 10,000 clinical isolates, WGS informed by known resistance gene variants was successful in predicting susceptibility to isoniazid, rifampicin, ethambutol, and pyrazinamide, with sensitivities of 97.1%, 97.5%, 94.6%, and 91.3%, respectively (78). As a direct clinical diagnostic, WGS has been proposed for TB susceptibility testing in low-incidence, high-resource settings based primarily on its more rapid time to results than conventional culture-based testing (which typically requires about 2 weeks after initial cultivation), alongside a slightly cheaper cost (79–81). In 2017, the United Kingdom adopted a policy of routine WGS for taxonomic and drug susceptibility testing of positive mycobacterial cultures. Subsequent retrospective analyses found partial success, noting the potential benefits of WGS but also the substantial lag in turnaround time, which is of particular concern regarding isoniazid resistance (82). Both cost and turnaround time would be expected to decrease as sequencing technology improves and is better incorporated into the clinical workflow. Notably, proof-of-concept studies in the Kyrgyz Republic and South Africa have demonstrated a potential role for *M. tuberculosis* WGS for resistance testing in low- and middle-income, high-burden countries (9, 83). Settings with a known high burden of MDR- and XDR-TB in particular may benefit from using WGS to identify and discontinue ineffective drugs, which frequently carry high toxicities for patients (9).

Beyond its role in diagnostics, sequencing the *M. tuberculosis* genome catalyzed the development of tests for *M. tuberculosis* drug resistance, notably the Xpert platform with MTB/XDR cartridges. Using microfluidics, this instrument amplifies *M. tuberculosis* genomic regions of interest and employs 10 molecular beacon probes to detect characteristic shifts in hybridization melting temperature. The assay identifies known resistance mutations to isoniazid, fluoroquinolones, and select second-line drugs directly from patient sputum or other clinical samples (8). Additionally, the test distinguishes between low- and high-level resistance mutations to isoniazid and fluoroquinolones, as well as cross-resistance to multiple second-line drugs (8). These genomic targets were gleaned from sequencing of many resistant *M. tuberculosis* isolates to define a list of the most common mutations. Early studies of the Xpert MTB/XDR test for clinical evaluation showed promising results, with 98% to 100% specificity for each of isoniazid, fluoroquinolones, ethionamide, amikacin, kanamycin, and capreomycin (84, 85). Sensitivity was highest for isoniazid and fluoroquinolones but low for ethionamide; this was attributed to the comparatively poorly characterized *ethA* resistance mutations and highlighted that molecular tests are limited by the genomic knowledge that underpins them (81, 84).

Unique among diagnostic methods, WGS can not only examine known resistance-associated polymorphisms but also predict novel variants associated with phenotypic resistance. Using principles including positive selection, convergent evolution, and non-synonymous to synonymous SNP ratios, several groups have built lists of genes and genomic regions that may represent unknown *M. tuberculosis* resistance mechanisms or help compensate for the fitness costs of co-occurring ones (28–30). Intriguingly, the prevalence of particular resistance mutations differs between lineages, suggesting that local *M. tuberculosis* strain demographics must also be considered when cataloging resistance mechanisms (86). The Comprehensive Resistance Prediction for Tuberculosis: an International Consortium (CRyPTIC) collaboration recently published a compendium of phenotype-validated *M. tuberculosis* WGS resistance data, a vital resource that will aid future susceptibility testing (31). The developing role of WGS in *M. tuberculosis* resistance testing has been explored in further detail in other work (87–89).

## Novel drugs

The past decade has seen a renewed effort toward the development of new antitubercular drugs, with the successful implementation of bedaquiline, delamanid, and pretomanid for MDR-TB (10, 11). At all stages of this process, from initial laboratory characterization to optimization of clinical regimens, WGS has proven essential. In the case of bedaquiline, a consistent *atpE* resistance mutant in vitro revealed that this drug uniquely targeted ATP synthesis and suggested that the compound could kill even dormant bacteria (90, 91). This *atpE* mutant was identified by genome sequencing of the model organism *M. smegmatis* and confirmed to likewise arise in resistant *M. tuberculosis* (90, 92). Testing across mycobacterial species demonstrated broad activity against many non-tuberculosis mycobacteria, except for *M. xenopi*, which was found by WGS to have a preexisting polymorphism at an *atpE* position known to confer resistance (90, 93). Subsequently, mutations in *Rv0678*, a repressor of the MmpS5/MmpL5 efflux machinery, were identified by WGS to cause cross-resistance to both bedaquiline and clofazimine (94). While reports differ, some sequencing studies argue that the off-target *Rv0678* polymorphisms are more frequent than *atpE* mutations in clinical isolates, at least in certain settings (95–97). An additional concern is the high prevalence of preexisting *Rv0678* mutations conferring measurably increased minimal inhibitory concentrations for bedaquiline and clofazimine in populations with no known prior use of either drug, since it had been assumed that bedaquiline would be effective in virtually all *M. tuberculosis* isolates early in its use (98).

Unlike the mechanism of action of bedaquiline, that of the nitroimidazoles pretomanid and delamanid was not readily apparent upon selection for resistance mutations. Initial characterizations identified resistance mutations in an F420-dependent enzyme, fgd1; however, other resistance mutations were soon reported from genome sequencing data (99, 100). Furthermore, despite evidence that pretomanid treatment impaired synthesis of ketomycolic acids, this was unlikely to be the mechanism of *M. tuberculosis* killing, as the drug was bactericidal even in non-replicating culture conditions when the bacterium would have little need to produce a cell wall component (99). Subsequent work found that fgd1 is one half of a pair of redox enzymes that acts cyclically on the F420 redox cofactor, the other of which is ddn, a nitroreductase that activates pretomanid to eventually form bactericidal nitric oxide (101, 102). Resistance to pretomanid can thus occur via mutations in either *fgd1* or *ddn*, or alternatively in the *fbiABCD* genes that synthesize F420 (95, 101, 103). Hence, the characterization by WGS of resistance polymorphisms to the nitroimidazole drugs led to the discovery of a mycobacterial metabolic pathway and a more plausible mechanism of killing: reactive nitrogen species production. In this way, genome sequencing can assign functions to unknown gene products. As with bedaquiline, mutations in the nitroimidazole-activating pathway genes have been found that predate drug use, some of which are predicted to confer resistance (104). Peculiarly, not all pretomanid-resistant mutants are cross-resistant to delamanid, so careful WGS of pretomanid-resistant, delamanid-susceptible isolates and vice versa is needed to fully characterize *M. tuberculosis* resistance patterns for the nitroimidazole class (103, 105).

## Epidemiology

### Outbreak tracing.

WGS can exploit polymorphisms in *M. tuberculosis* genomic isolates for robust epidemiological tracing regardless of whether the polymorphisms have a known functional role. On the local scale, genome sequencing can resolve TB outbreaks down to individual-level spreading events, enabling the identification of social and socioeconomic factors contributing to transmission (5–7). Older methods such as MIRU-VNTR typing may be sufficient for initial surveillance, though WGS allows for more precise tracing of spread through a community (5, 6). A systematic comparison of MIRU-VNTR and WGS found that MIRU-VNTR can help exclude transmission events on the basis of differences, but it is much less accurate for positively predicting transmission by strain relatedness (106). In addition, MIRU-VNTR predictive efficacy varies by *M. tuberculosis* lineage, with L2 in particular known to have poor discrimination due to evolutionary convergence in its VNTR regions (106, 107). Aggregate WGS surveillance data are more consistently accurate and can be systematically analyzed for variables such as *M. tuberculosis* sublineage and frequency of resistance polymorphisms, although currently the majority of *M. tuberculosis* isolates are not sequenced (49). To potentially decrease the computational load of screening the entire *M. tuberculosis* genome, a minimal set of SNPs has been proposed as a barcode to stratify *M. tuberculosis* sublineages as a rapid surveillance tool (55, 56). While widespread use of WGS for surveillance is limited by expense and expertise, particularly in high-burden settings, access may expand in the future as cost improves (108).

### Recent versus remote transmission of TB.

Prior to the 1990s, the prevailing belief held that the majority of active TB cases were the result of endogenous reactivation of latent TB infection (LTBI) that was acquired remotely in time (109). However, in the early 1990s, IS6110 molecular typing of large *M. tuberculosis* strain banks collected contemporaneously from active TB cases across US cities yielded the unexpected result that approximately 40% of strains matched another isolate in the collection (110–112). In some instances, it was possible to establish epidemiological links between individuals with matching strains (112). This suggested that recently transmitted TB could lead to active TB disease in a short period of time rather than requiring reactivation of LTBI acquired years or decades earlier. Such findings, based on *M. tuberculosis* genomic data, have prompted some to make the controversial argument that most infected individuals are cleared of bacteria after two years and are therefore no longer at risk for reactivation of LTBI (113, 114). The issue has important ramifications for how intensively public health organizations should seek out individuals with LTBI to provide secondary prevention therapy with isoniazid for 9 months or a shorter-course regimen (115, 116). Indeed, there has been a trend in the United States to limit LTBI testing by tuberculin skin test or interferon-γ release assay to select high-risk groups (117). Interestingly, recent work using WGS to address the matter of recent versus remote infection found that longer latency times do not necessarily correlate with SNP distance — a finding that calls into question the reliability of genomic determinants in estimating the recency of infection (118). Additional studies using high sequencing depth to query *M. tuberculosis* mutation rates in human patients, particularly during paucibacillary states, may refine the relationship between the rate of *M. tuberculosis* mutation acquisition and latency time to enhance epidemiological tracking tools.

### WGS to determine mutation rates in latent versus active TB.

In addition to studying the emergence of particular variants of interest across broad geographies, WGS offers the capability of examining mutational changes in the *M. tuberculosis* population within a single individual. Studies of *M. tuberculosis* genomes from patients receiving TB treatment have consistently found heterogeneity in bacterial populations, with drug resistance driven by mutants that are initially detectable only at low levels (32–34). Most population heterogeneity was driven by SNPs with less than 20% frequency, and indeed a population prevalence of merely 19% was estimated to be the threshold required for a drug resistance variant to proceed toward fixation (33, 34). The most common variation at the nucleotide level was the GC > AT transition, predicted to be a product of oxidative damage faced by the bacterium in the host macrophage, which was first noted in prior nonhuman primate data (34, 119). This latter model also found that *M. tuberculosis* mutation rates remained similar in active, latent, and reactivated TB disease, a striking finding given that LTBI was previously believed to reflect low numbers of bacilli with little or no replication and thus a negligible risk of mutation. Such findings raise the concern that the use of isoniazid monotherapy for 9 months as secondary prevention for LTBI would potentially risk isoniazid resistance (119–121). Consistent with the threat of resistance due to inadequate treatment, *M. tuberculosis* WGS data during active TB treatment showed a risk of excessive drug resistance mutations in patients receiving fewer than 4 effective drugs (33). These results collectively underscore the high threshold for adequate antimicrobial pressure, below which even rare *M. tuberculosis* resistance mutants can ultimately reach fixation within a patient. Additionally, there is some evidence linking high intra-host *M. tuberculosis* diversity to worse TB severity metrics (122). It remains to be seen whether the use of WGS to query *M. tuberculosis* heterogeneity within a patient has a role as a disease prognostic marker or for monitoring the development of resistance.

Epidemiological studies tracking *M. tuberculosis* strains and lineages are greatly aided, if not outright enabled, by the slow mutation rate of the organism. In vitro, the *M. tuberculosis* genome is remarkably stable, with mutations mainly at the level of individual bases; polymorphisms due to polymerase errors in repetitive microsatellite regions are comparatively rarer than would be expected by chance, and large-scale chromosomal rearrangements are almost entirely absent (44, 123). Complicating the earlier finding of a low, constant mutation rate in nonhuman primates, subsequent studies using deeper sequencing in human patients found substantial population heterogeneity, suggesting that the in vivo mutation rate may be higher than that observed in vitro (32–34, 119). Additionally, the Beijing sublineage of *M. tuberculosis* has been proposed to acquire antimicrobial resistance more rapidly owing to a faster mutation rate, though this remains controversial (123–127).

### Molecular epidemiology of drug-resistance emergence.

Public health interventions that target the emergence of drug-resistant TB are severely needed. Successful treatment outcomes are achieved in just under 60% of patients with MDR-TB globally and only 40% of patients with XDR-TB, and some cohorts experience much less success in the latter case (1, 128, 129). Algorithms that calculate the most parsimonious path to a common ancestor strain enable so-called molecular clock analyses that predict the order in which SNPs, including those conferring drug resistance, arise within a pool of clinical isolates. When applied to WGS data of contemporary isolates from the KwaZulu-Natal province of South Africa, these tools predicted that the evolution of current XDR strains in South Africa began in the mid-1950s, with isoniazid mono-resistance occurring first, followed by sequential accumulation of additional drug resistance mutations over time (35). In addition, this work predicted a rapid acquisition of rifampicin resistance polymorphisms once *M. tuberculosis* acquired isoniazid mono-resistance and found both preexisting and novel mutations likely to compensate for the fitness costs of acquiring rifampicin resistance (35). Broadening this WGS approach to encompass a global range of *M. tuberculosis* isolates revealed a similar finding: that the Ser315Thr mutation in *katG* conferring isoniazid resistance occurred prior to rifampicin resistance in over 90% of measured instances, regardless of time period or geography (36). Notably, isoniazid mono-resistance is not detected by the Xpert MTB/RIF cartridge, a broadly used molecular test to both diagnose TB and screen for common rifampicin resistance mutations (130). As a result, TB cases found to be rifampicin-susceptible by Xpert receive standard TB therapy using only two drugs — isoniazid and rifampicin — in the 4-month continuation phase (120, 121). Thus, undetected isoniazid–mono-resistant cases may receive the equivalent of unprotected rifampicin monotherapy, thereby risking rapid loss of rifampicin susceptibility.

## BCG

**Figure 3:**
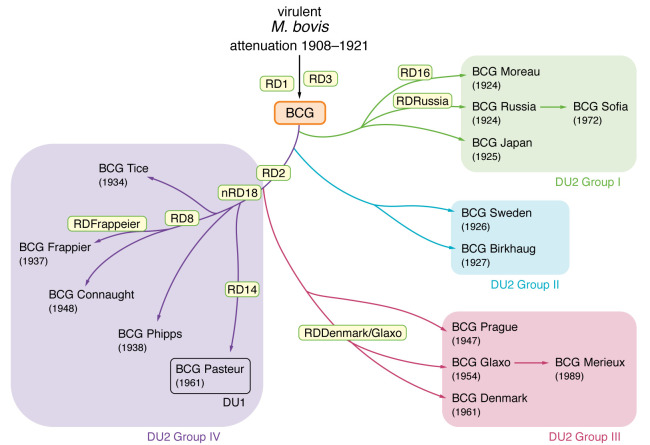
Genealogy of BCG daughter strains. Evolutionary trajectory depicting selected regions of difference (RDs) that define the modern BCG vaccine strains in relation to the parental virulent *M. bovis*. Strains are clustered by the presence of tandem duplications (DU1 and DU2). Adapted with permission from *Vaccines* (173).

The only currently approved and effective TB vaccine is BCG, a live, attenuated derivative of *M. bovis* developed by serial passage in vitro over a 13-year period (131). This vaccine strain was rapidly distributed at global scale and maintained as numerous daughter strains (Figure 3) that underwent decades of further passages prior to the advent of bacterial storage technology. Consequently, these various BCG strains contain overlapping but distinct subsets of RDs from the parental *M.* *bovis*. Chief among these, RD1 is absent from all BCG strains but present in virulent *M.* *bovis* isolates (132). RD1 deletions in three distinct *M. tuberculosis* strains and *M. bovis* caused avirulence, which was restored by complementation, validating RD1 as the primary attenuating mutation in BCG (25). Beyond RD1, however, the BCG daughter strains are not uniform for the remaining RDs and possess additional polymorphisms beyond the RD deletions (12, 15, 134). Numerous studies have examined the TB protection conferred by the global BCG repertoire in animal models and consistently found measurable differences between BCG strains (12–14, 135). Additionally, BCG strains differ in their predicted T cell epitopes, gene expression, and virulence toward immune-deficient mice, among other properties (12, 14, 136, 137). This variability extends to comparative studies of differing BCG strains in infants, as assessed by immune metrics (138–141). It is not clear at present whether these findings should influence the choice of BCG vaccine strain in different settings, particularly because of the infrastructural and ethical challenges associated with such a decision. As genome sequencing uncovers higher-resolution information regarding the global diversity of BCG strains, it is challenging to identify a consistent impact of these polymorphisms on clinical efficacy. However, this body of work clearly justifies additional consideration in BCG strain selection for laboratory studies, particularly if the aim is to generate an improved TB vaccine (142).

There remain additional opportunities to make further use of WGS related to the BCG vaccine. Surveillance of BCG seed lots using deep sequencing has uncovered heterogeneity between and even within lots (143, 144). In select instances, variants were identified in known virulence-promoting genes or in secretory loci that may alter the antigenic profile of the vaccine (144). BCG diversity extends far beyond the large genomic deletions classified as RDs, and many variants similarly impact cell wall and secretory factors predicted to interact with the host (12, 137). Additional data regarding BCG protective efficacy and adverse outcomes may uncover determinants of mycobacterial immunogenicity if these vaccine properties can be consistently correlated to BCG genotype.

## PE/PPE proteins

While most proteins in the *M. tuberculosis* genome could be classified into known or putative functions once their sequences were obtained, there remained 16% without similarity to previously known proteins (4). Among these are the PE and PPE families, named for the conserved Pro-Glu and Pro-Pro-Glu amino acids near their N-termini, which were uniquely discovered by *M. tuberculosis* WGS (4). Phylogenetic analysis of these families revealed a massive duplication of these proteins in pathogenic mycobacteria, especially the PE_PGRS (polymorphic GC-rich repeat sequence) and the PPE-MPTR (major polymorphic tandem repeat) subfamilies (145). Notably, this expansion was predated by the duplication of the ESX-2 secretion system to yield ESX-5, the most recently evolved type VII secretion system in *M. tuberculosis* (145, 146). The ESX-5 system was subsequently found to secrete PE/PPE proteins via a conserved secretion signal on the C-terminus of the PE and PPE domains, potentially rendering ESX-5 responsible for the export of approximately 150 members of the largely uncharacterized PE/PPE families (147, 148). Saturating transposon mutagenesis demonstrated that the vast majority of PE/PPE proteins are nonessential for *M. tuberculosis* growth in vitro, suggesting instead a role during infection (149–151). Indeed, an *M. tuberculosis* strain deficient for ESX-5 showed attenuation in mice, and PE/PPE proteins are highly secreted in *M. tuberculosis* animal models and human infections (152–156). Intriguingly, mutation at the *ppe38* locus impairs secretion of many PE_PGRS and PPE-MPTR proteins but increases virulence, which is notable because the hypervirulent Beijing sublineage possesses this mutation (157, 158). As a class, PE/PPE genes show frequent polymorphisms and recombination events across clinical strains, which may impact their efficacy as antigens and putative vaccine candidates; the PPE18 component of the M72/AS01E vaccine has over 60 known non-synonymous SNPs, for example (159, 160). Clear functions have been identified for several family members, but most remain weakly characterized (161, 162). Relatedly, reads encompassing these genes are often omitted from *M. tuberculosis* WGS and similar studies owing to the high similarity between family members. Other reviews document the growing understanding of the roles of PE/PPE proteins during *M. tuberculosis* infection (163–165).

## Essentiality and vulnerability

With the immense global need for novel antitubercular drugs, the *M. tuberculosis* genome was probed to uncover targetable pathways almost as soon as it was reported in 1998. Transposon sequencing (TnSeq) uses mobile genetic elements to create loss-of-function insertions across the bacterial genome. Large pooled libraries of *M. tuberculosis* strains with single transposon insertions are then evaluated in different environmental conditions to identify mutants that decrease in abundance over time, suggesting fitness defects conferred by disruption of individual genes. This approach identified genes essential for growth in vitro, a candidate list for potential therapeutic intervention (149–151). To identify *M. tuberculosis* genes essential in vivo and determine the subset of these factors that interact with host genotype, an *M. tuberculosis* transposon library was infected into the Collaborative Cross panel of outbred mice (39, 166). Notably, expanding the range of mouse genotypes more than doubled the number of *M. tuberculosis* genes implicated in infecting at least one host background (39). This study made exceptional use of WGS technology in a rigorous attempt to model interindividual diversity during *M. tuberculosis* infection.

TnSeq is valuable in building breadth of understanding about the *M. tuberculosis* genome, but an ideal ranking scheme would quantitatively prioritize candidate essential genes where pharmacological inhibition would be most lethal for the pathogen. This is the basis of “vulnerability” studies, which have demonstrated that certain bacterial pathways are highly susceptible to low levels of depletion while others are durable even to near-total knockdown (167, 168). Recently, this concept has incorporated CRISPR interference (CRISPRi), which uses a deactivated version of the Cas machinery that acts as a sequence-specific transcriptional repressor. This targeted approach allows the tuning of expression levels across almost all essential genes of *M. tuberculosis* (37). Adapting this approach to a chemical-genetic design demonstrated known and new resistance pathways for first- and second-line TB drugs, including cell envelope and stress response pathways that caused broad cross-resistance to many compounds (38). Most excitingly, this study identified a new *whiB7* polymorphism that caused macrolide hypersensitivity in a particular *M. tuberculosis* L1 sublineage and further showed that mice infected with an L1 strain responded well to clarithromycin therapy in a short-term model. Addition of a macrolide to standard TB therapy might accelerate the treatment time for TB patients in Southeast Asia who are infected by L1 strains with this polymorphism (38). This recently reported example of unexpected macrolide susceptibility in an appreciable number of TB strains in Asia represents a promising example of how *M. tuberculosis* genomics can yield clinical solutions.

## Conclusions and lingering questions

The *M. tuberculosis* genome sequence and subsequent *M. tuberculosis* WGS efforts have impacted nearly every aspect of the TB field. Many therapeutic innovations, including the newest generation of antitubercular drugs, rely on this technology for an improved ability to identify therapeutic targets and track resistance mechanisms (81, 95, 96). Diagnostic advancements, such as the Xpert MTB/XDR assay, likewise hinge on the growing power of *M. tuberculosis* genomics (8, 84, 85). Epidemiological surveillance of TB has been extensively informed by WGS both to track spread within communities and to follow *M. tuberculosis* lineages across broader patterns of geographic and temporal spread (6, 36, 49). Innovations stemming from the *M. tuberculosis* genome sequence will likely further broaden in the coming years, as findings from patient-level investigations and innovative whole-genome bacteriological designs are pursued for translational application (32–34, 37, 38).

While the genome sequence is a valuable road map, it is important to note that causality of phenotypes cannot be concluded from DNA sequences but rather requires gene transfer technologies based on plasmids, phage elements, and gene recombination tools (16–27). The many understudied variants seen in *M. tuberculosis* clinical isolates may provide additional insight into bacterial factors that promote host infection (31, 86, 159, 160). Relatedly, there is growing interest in associating bacterial and human genome variations to demonstrate bidirectional selective pressure exerted by the pathogen and the host immune system on one another (53, 54, 68, 71–74). We note as well several examples in which WGS has identified preexisting drug resistance or susceptibility in populations, though the appropriate use of this technology to inform region-specific regimen selection must be further considered (38, 98, 104). In addition, the adoption of a persistence phenotype by *M. tuberculosis*, which allows the organism to survive in the host for extreme durations, remains an area of great interest. Understanding how gene expression regulates the persistence phenotype may lead to novel approaches to allow drugs and the immune response to sterilize *M. tuberculosis* infections (77, 169–171).

## Version 1 10/02/2023

Electronic publication
